# Transcriptome Analysis of *Houttuynia cordata* Thunb. by Illumina Paired-End RNA Sequencing and SSR Marker Discovery

**DOI:** 10.1371/journal.pone.0084105

**Published:** 2014-01-02

**Authors:** Lin Wei, Shenghua Li, Shenggui Liu, Anna He, Dan Wang, Jie Wang, Yulian Tang, Xianjin Wu

**Affiliations:** 1 The College of Life Science, Huaihua University, Huaihua, China; 2 Key Laboratory of Hunan Province for Study and Utilization of Ethnic Medicinal Plant Resources, Huaihua, China; 3 Key Laboratory of Hunan Higher Education for Hunan-Western Medicinal Plant and Ethnobotany, Huaihua, China; 4 The College of Life Science, Hunan University, Changsha, China; Auburn University, United States of America

## Abstract

**Background:**

*Houttuynia cordata* Thunb. is an important traditional medical herb in China and other Asian countries, with high medicinal and economic value. However, a lack of available genomic information has become a limitation for research on this species. Thus, we carried out high-throughput transcriptomic sequencing of *H. cordata* to generate an enormous transcriptome sequence dataset for gene discovery and molecular marker development.

**Principal Findings:**

Illumina paired-end sequencing technology produced over 56 million sequencing reads from *H. cordata* mRNA. Subsequent de novo assembly yielded 63,954 unigenes, 39,982 (62.52%) and 26,122 (40.84%) of which had significant similarity to proteins in the NCBI nonredundant protein and Swiss-Prot databases (E-value <10^−5^), respectively. Of these annotated unigenes, 30,131 and 15,363 unigenes were assigned to gene ontology categories and clusters of orthologous groups, respectively. In addition, 24,434 (38.21%) unigenes were mapped onto 128 pathways using the KEGG pathway database and 17,964 (44.93%) unigenes showed homology to *Vitis vinifera* (Vitaceae) genes in BLASTx analysis. Furthermore, 4,800 cDNA SSRs were identified as potential molecular markers. Fifty primer pairs were randomly selected to detect polymorphism among 30 samples of *H. cordata*; 43 (86%) produced fragments of expected size, suggesting that the unigenes were suitable for specific primer design and of high quality, and the SSR marker could be widely used in marker-assisted selection and molecular breeding of *H. cordata* in the future.

**Conclusions:**

This is the first application of Illumina paired-end sequencing technology to investigate the whole transcriptome of *H. cordata* and to assemble RNA-seq reads without a reference genome. These data should help researchers investigating the evolution and biological processes of this species. The SSR markers developed can be used for construction of high-resolution genetic linkage maps and for gene-based association analyses in *H. cordata*. This work will enable future functional genomic research and research into the distinctive active constituents of this genus.

## Introduction

Saururaceae, a member of the paleoherbs, is an ancient family with six species in four genera, *Anemopsis*, *Gymnotheca*, *Houttuynia* and *Saururus* [1]. *Houttuynia cordata* Thunb. (Yuxingcao in Chinese) is the only species in the genus *Houttuynia* [2], [3]. It is distributed mainly in the central, southeastern and southwestern regions of China, and extends to Japan, Korea and Southeast Asia, where it grows in moist, shady places [4]. *H. cordata* is an important traditional medical herb native to China and other Asian countries [5], [6]. It plays a unique role in improving the immune system of patients with severe acute respiratory syndrome (SARS) [7], [8]. Extracts of *H. cordata* have diverse pharmacological effects including anticestodal [9], antibacterial [10], [11], antiviral [12]–[15], anticancer [16], [17], antioxidant [18], [19], antiallergenic [20], [21], anti-inflammatory [22]–[24], antimutagenic [18] and anti-obesity [25] activities. *H. cordata* is also consumed as a vegetable in China for its special aroma. Although *H. cordata* is of high medicinal and nutritional value, there are no genomic resources for this non-model genus. This lack of genomic information has become a limitation for extensive and intensive research on this important traditional medical herb.

Previous studies on this plant have mainly focused on cultivation techniques [26], [27], its physiological and biochemical properties [28], [29], its genetic relationships and the diversity among *H. cordata* germplasm collections from different places [4], [30], [31], and its pharmacological effects [7]–[25]. To date, few gene sequences or novel genes have been reported on this species, although much effort has been devoted to cloning key genes. RNA-Seq, which is based on next generation sequencing, is a high throughput technology that has great advantages in examining the fine structure of a transcriptome [32]. When no genome sequence is available, transcriptome sequencing provides an effective way to obtain large amounts of sequence data [33]. RNA-Seq has been widely used in many organisms to obtain mass sequence data for transcriptional analysis, gene discovery and molecular marker development [34]–[36]. The genetic relationships and diversity among *H. cordata* germplasm collections have been investigated mostly using AFLP [37], ISSR [4], PCR-RFLP [30] and RAPD markers [31]. No simple sequence repeat (SSR) markers have been reported in *H. cordata*. Compared with other types of molecular markers, SSR markers have many advantages, such as simplicity, effectiveness, abundance, hypervariability, reproducibility, codominant inheritance, and extensive genomic coverage [38]. Because of the lack of effective molecular markers, marker-assisted selection and molecular breeding of *H. cordata* has lagged behind other medicinal plants such as *Panax notoginseng* (Burkill) F.H.Chen [39], *Gastrodieae elata* Blume [40], and *Glycyrrhiza uralensis* Fisch. [41]. Thus, a rapid, low-cost and effective approach is required to develop SSRs molecular markers for *H. cordata*.

In this study, we applied the next-generation massively parallel sequencing technique (Illumina HiSeq 2000) to the sequencing and analysis of the complete *H. cordata* transcriptome for the first time. We sampled the pooled transcriptomes of flower, leaf, stem and rhizome tissues of *H. cordata* and used Illumina paired-end sequencing technology to generate a large-scale EST database and develop a set of SSR markers. These results provide a very useful genomic resource for research on *H. cordata* in the future.

## Results

### Illumina paired-end sequencing and de novo assembly

To obtain a global overview of the *H. cordata* transcriptome and gene activity at the nucleotide resolution, RNA was extracted from four different *H. cordata* tissues including the rhizome, stem, leaf and flower, and mixed at equivalent concentrations. Sequencing was performed on an Illumina HiSeq2000 genome analyzer. Each sequencing pass can yield two 90-bp independent reads from either end of a DNA fragment. In all, 56,668,324 sequence reads were generated, of which 51,973,070 were of acceptable quality after cleaning the low-quality reads (Table 1), then we used the Trinity short reads assembly program to assemble the reads for non-redundant consensus [42]. The sequence data were deposited in the NCBI Sequence Read Archive (http://www.ncbi.nlm.nih.gov/Traces/sra) under the accession number SRR871486.

**Table 1:** Summary of sequencing output statistics.

Samples	Total Raw Reads	Total Clean Reads	Total Clean Nucleotides*	Q20%	N%	GC%
*H. cordata*	56,668,324	51,973,070	46,77,576,300	97.83%	0.00%	50.62%

*Total Clean Nucleotides  =  Total Clean Reads1×Read1 size+Total Clean Reads2×Read2 size.

**Figure 1:**
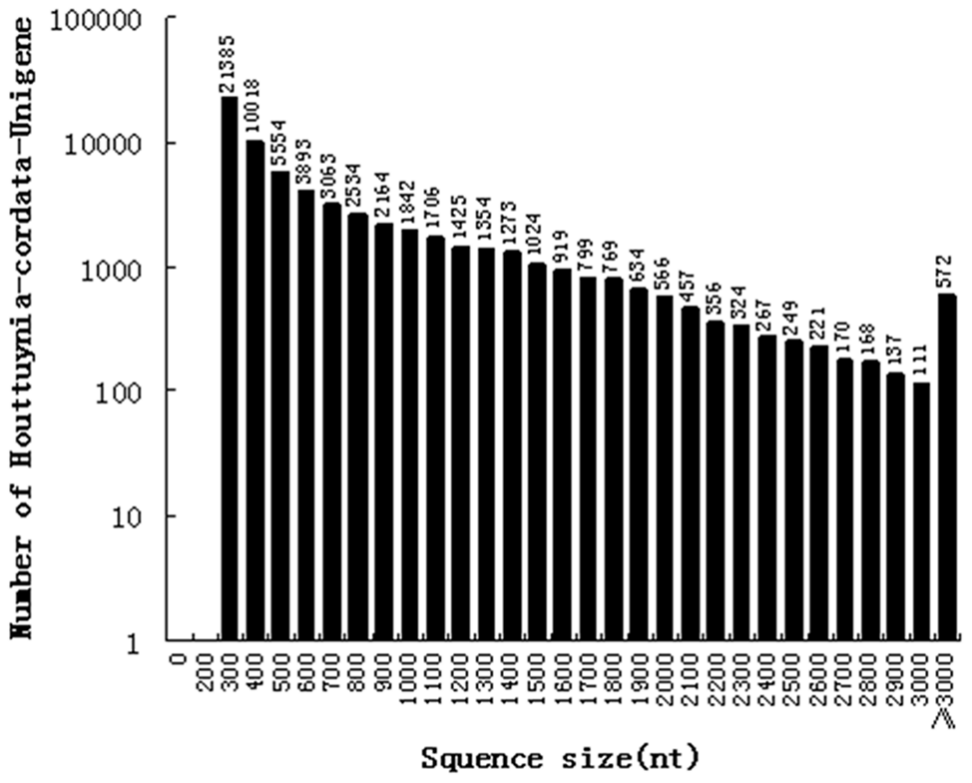
Length distribution of *H. cordata* Unigenes.

De novo assembly yielded 63,954 unigenes with median length of 1051 bp and a total length of over 43.395 Mb. The lengths of the assembled unigenes ranged from 200 to 8,507 bp. There were 36, 957 unigenes (57.79%) with lengths varying from 201 to 500 bp, 13,496 unigenes (21.10%) in the length range 501–1000 bp, 6,782 unigenes (10.60%) with lengths varying from 1001 to 1500 bp, 3,687 unigenes (5.77%) with lengths varying from 1501 to 2000 bp, and 3,032 unigenes (4.74%) more than 2000 bp long (Fig. 1).

### Sequence annotation

**Figure 2:**
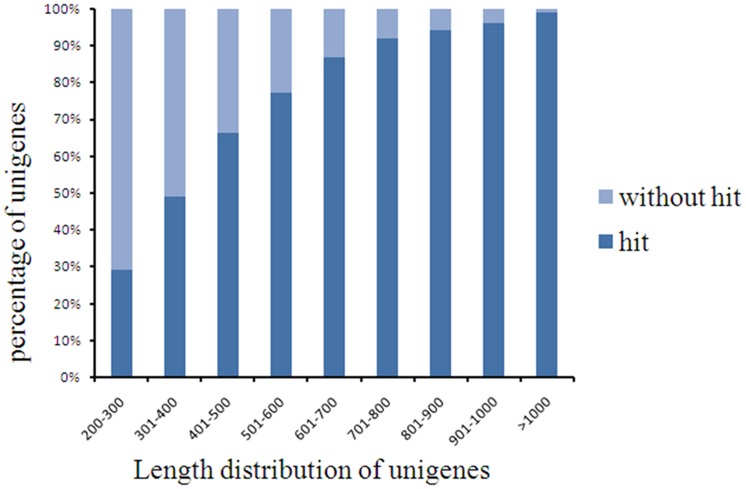
Comparison of unigene length with or without hits.

Several complementary methods were used to annotate the assembled sequences. First, the assembled sequences were queried against the National Center for Biotechnology Information (NCBI) nonredundant protein (Nr) and Swiss-Prot protein databases using BLASTx to search for similar sequences (E-value <10^−5^). Of the 63,954 assembled sequences, 39,982 (62.52%) showed homology to sequences in the Nr database (Table S1), while 26,122 (40.84%) unigenes had homology to proteins in the Swiss-Prot database. In addition, 99.06% of the unigenes over 1,000 bp in length showed homologous matches, whereas only 29.28% of the unigenes shorter than 300 bp showed matches (Fig. 2). The 2,624 unigenes that had no matches in either the Nr or Swiss-Prot databases were subjected to gene prediction analysis using ESTScan (Version3.0.2) [43]. In total, 42,785 unigenes were detected by homology analysis using the Nr and Swiss-Prot databases or ESTScan prediction.

**Figure 3:**
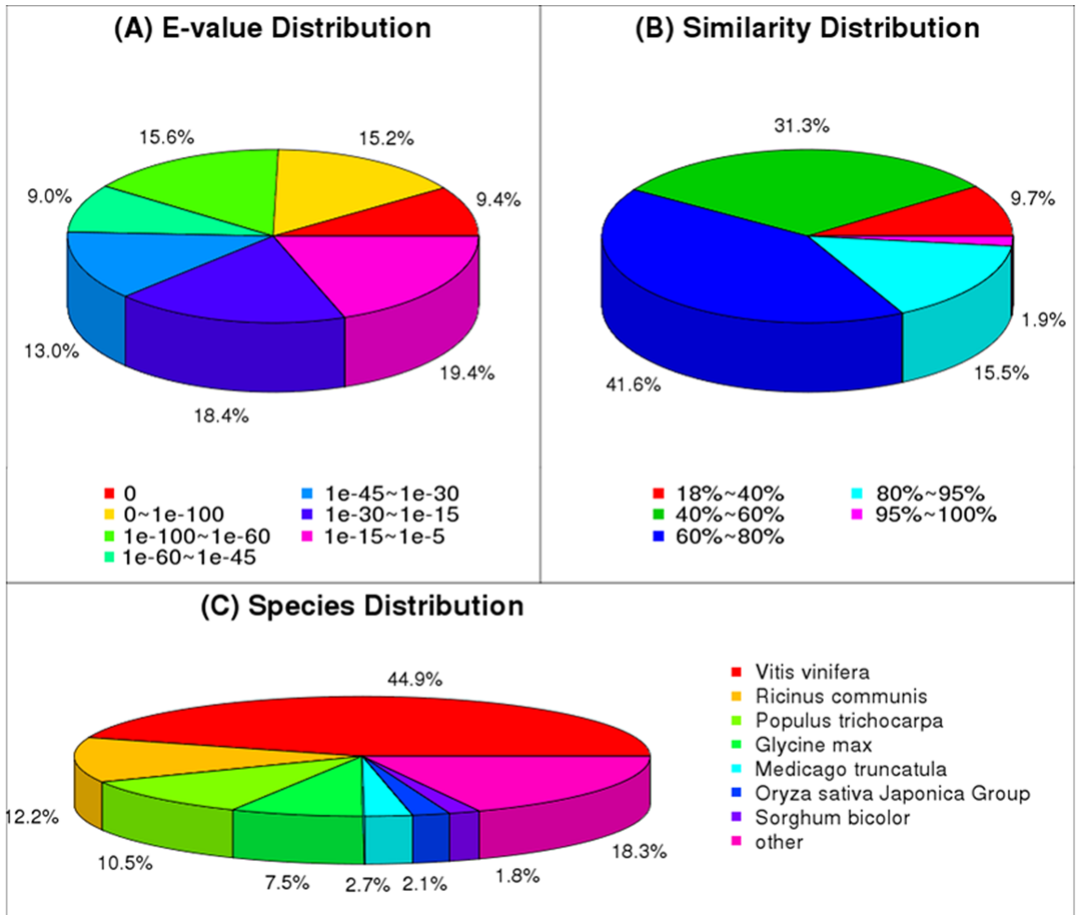
Characteristics of homology search of Illumina sequences against the nr database. (A) E-value distribution of BLAST hits for each unique sequence with a cut-off E-value of 1.0E-5. (B) Similarity distribution of the top BLAST hits for each sequence. (C) Species distribution is shown as a percentage of the total homologous sequences with an E-value of at least 1.0E-5.

The E-value distribution of the top hits in the Nr database showed that 62.2% of the mapped sequences had strong homology (E-value <10^−30^) and 50.8% had very strong homology (E-value <10^−45^) to available plant sequences, whereas 37.8% of the homologous sequences had E-values in the range 10^−5^ to 10^−30^ (Fig. 3A). The similarity distribution of sequences database showed that 3,880 (9.70%), 12,513 (31.30%), 16,642 (41.62%), 6,198 (15.50%) and 749 (1.87%) sequences were 18–40%, 41–60%, 61–80%, 81–95% and 95–100% similar, respectively (Fig. 3B). In terms of species distribution, 44.93%, 12.16%, 10.51%, 7.53%, 2.26%, 2.21% and 1.75% of the distinct sequences had matches to sequences from *Vitis vinifera* (Vitaceae), *Ricinus communis* (Euphorbiaceae), *Populus trichocarpa* (Salicaceae), *Glycine max*, *Medicago truncatula* (Leguminosae), *Oryza sativa* Japonica Group, and *Sorghum bicolor* (Gramineae), respectively. And 18.35% of the distinct sequences had matches to sequences from ‘other’ species. (Fig. 3C).

**Figure 4:**
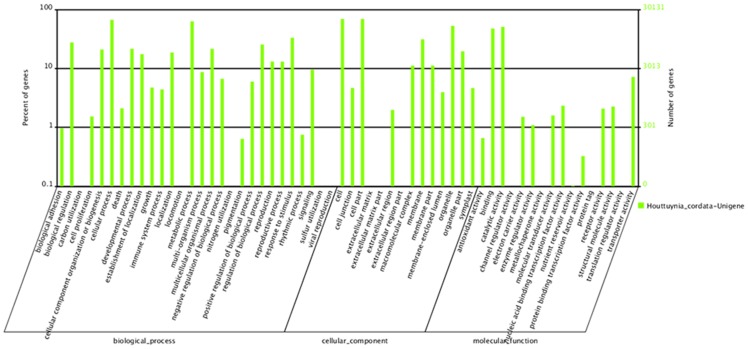
Gene ontology classification of assembled unigenes.

Based on Nr annotation, 30,131 unigenes were assigned gene ontology (GO) terms. The sequences that belonged to the biological process, cellular component, and molecular function clusters were categorized into 46 functional groups (Fig. 4). ‘Cellular processes’ and ‘metabolic processes’, ‘cell’ and ‘cell part’, ‘antioxidant activity’ and ‘binding’ were the dominant groups among the three main categories (biological process, cellular component and molecular function), respectively. However, we did not find any genes in the clusters ‘carbon utilization’, ‘locomotion’, ‘nitrogen utilization’, ‘sulfur utilization’, ‘viral reproduction’, ‘extracellular matrix’, ‘extracellular matrix part’, ‘extracellular region part’, ‘channel regulator activity’, ‘metallochaperone activity’, ‘protein tag’ or ‘translation regulator activity’ (Fig. 4).

**Figure 5:**
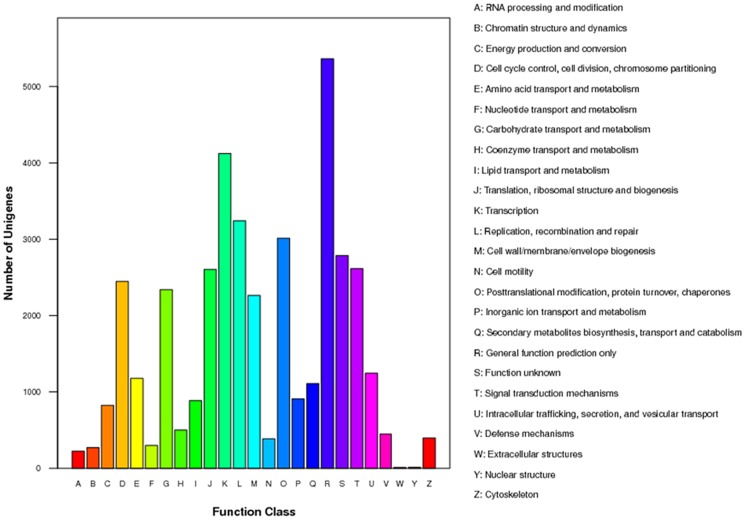
Clusters of orthologous groups (COG) classification.

In addition, all unigenes were subjected to a search against the Cluster of Orthologous Groups (COG) database for functional prediction and classification. Overall, 15,363 of the 39,982 sequences showing Nr hits were assigned COG classifications. The COG-annotated putative proteins were functionally classified into at least 25 molecular families including cellular structure, biochemistry metabolism, molecular processing, and signal transduction (Fig. 5).

The cluster for ‘general function prediction only’ (5,362, 13.58%) represented the largest group, followed by transcription (4,124, 10.44%), replication, recombination and repair (3,242, 8.21%), posttranslational modification, protein turnover and chaperones (3,014, 7.63%), function unknown (2,787, 7.06%), signal transduction mechanisms (2,614, 6.62%), translation, ribosomal structure and biogenesis (2,603, 6.59%), cell cycle control, cell division, chromosome partitioning (2,446, 6.19%), carbohydrate transport and metabolism (2,340, 5.93%), cell wall/membrane/envelope biogenesis (2,263, 5.73%), intracellular trafficking, secretion, and vesicular transport (1,244,3.15%), amino acid transport and metabolism (1,177,2.98%), secondary metabolites biosynthesis, transport and catabolism (1,109, 2.81%), inorganic ion transport and metabolism (909, 2.30%), lipid transport and metabolism (887, 2.25%), energy production and conversion (823, 2.08%), coenzyme transport and metabolism (501, 1.27%), defense mechanisms (447, 1.13%), cytoskeleton cell (398, 1.00%), motility (385, 0.98%), nucleotide transport and metabolism (298, 0.75%), chromatin structure and dynamics (270, 0.68%) and RNA processing and modification (222, 0.56%), whereas only a few unigenes were assigned to nuclear structure and extracellular structure (13 and 11 unigenes, respectively). The COG function classification of *H. cordata* unigenes is shown in Fig. 5.

### Metabolic pathway assignment by KEGG analysis

A total of 24,434 assembled sequences were associated with 128 predicted KEGG metabolic pathways. The number of sequences ranged from 3 to 6,718. The top 20 pathways with the greatest number of sequences are shown in Table 2. The greatest number of transcripts was found in the metabolic pathways. The top 10 metabolic pathways were: glycerophospholipid metabolism (1,974), ether lipid metabolism (1,853), starch and sucrose metabolism (869), purine metabolism (697), pyrimidine metabolism (637), phenylpropanoid biosynthesis (381), oxidative phosphorylation (298), amino sugar and nucleotide sugar metabolism (295), glycolysis/gluconeogenesis (267), and flavonoid biosynthesis (214) (Table S2).

**Table 2:** The top 20 pathways with highest sequence numbers.

Number	Pathway	All genes with pathway annotation (24434)	Pathway ID
1	Metabolic pathways	6,718 (27.49%)	ko01100
2	Biosynthesis of secondary metabolites	2,448 (10.02%)	ko01110
3	Endocytosis	2,112 (8.64%)	ko04144
4	Glycerophospholipid metabolism	1,974 (8.08%)	ko00564
5	Ether lipid metabolism	1,853 (7.58%)	ko00565
6	Plant-pathogen interaction	1,319 (5.4%)	ko04626
7	Plant hormone signal transduction	1,111 (4.55%)	ko04075
8	RNA transport	1,058 (4.33%)	ko03013
9	Spliceosome	894 (3.66%)	ko03040
10	Starch and sucrose metabolism	869 (3.56%)	ko00500
11	Purine metabolism	697 (2.85%)	ko00230
12	mRNA surveillance pathway	665 (2.72%)	ko03015
13	Pyrimidine metabolism	637 (2.61%)	ko00240
14	Protein processing in endoplasmic reticulum	602 (2.46%)	ko04141
15	Pentose and glucuronate interconversions	535 (2.19%)	ko00040
16	Ubiquitin mediated proteolysis	503 (2.06%)	ko04120
17	Ribosome	462 (1.89%)	ko03010
18	RNA polymerase	431 (1.76%)	ko03020
19	Ribosome biogenesis in eukaryotes	426 (1.74%)	ko03008
20	RNA degradation	426 (1.74%)	ko03018

### Development and characterization of SSR markers

For further assessment of the assembly quality and development of new molecular markers, all 63,954 unigenes generated in this study were used to mine potential microsatellites, which were defined as di- to hexa-nucleotide SSRs with a minimum of four repetitions for all motifs. The SSRs that were only located in one single read had been eliminated. Using the MIcroSAtellite (MISA, http://pgrc.ipk-gatersleben.de/misa/) tool, a total of 4,800 potential SSRs were identified in 4,413 unigenes, 357 of which contained more than one SSR; 164 SSRs were present in compound form (Table 3).

**Table 3:** Summary of SSR searching results.

Searching Item	Numbers
Total number of sequences examined	63,954
Total size of examined sequences (bp)	43,395,361
Total number of identified SSRs	4,800
Number of SSR containing sequences	4,413
Number of sequences containing more than 1 SSR	357
Number of SSRs present in compound formation	164
Mono- nucleotide	1,313
Di-nucleotide	1,278
Tri-nucleotide	1,994
Tetra-nucleotide	39
Penta-nucleotide	66
Hexa-nucleotide	110

The frequency, type and distribution of the 4,800 potential SSRs were also analyzed in this study. The compilation of all SSRs revealed that, on average, one SSR could be found every 9.04 kb in the unigenes. Among the 4,800 SSRs, tri-nucleotide repeat motifs were the most abundant type (1,994, 41.54%), followed by mono- (1,313, 27.35%), di- (1,278, 26.63%), hexa- (110, 2.29%), penta- (66, 1.38%) and tetra-nucleotide (39, 0.81%) repeat motifs. The mono- to hexa-nucleotide motifs were further analyzed for SSR repeat numbers (Table 4).

**Table 4:** Length distribution of SSRs based on the number of repeaters.

Number of repeaters	mono-	Di-	Tri-	Tetra-	Penta-	Hexa-	Total
4	-	-	-	-	57	94	151
5	-	-	1,275	34	9	10	1,328
6	-	446	481	5	0	2	934
7	-	284	208	0	0	3	498
8	-	202	21	0	0	0	223
9	-	145	3	0	0	0	148
10	-	119	0	0	0	0	119
11	-	78	2	0	0	0	80
12	604	4	0	0	0	0	608
13	305	0	1	0	0	0	306
14	169	0	0	0	0	0	169
15	108	0	0	0	0	0	108
16	38	0	1	0	0	1	40
17	21	0	0	0	0	0	21
18	3	0	1	0	0	0	4
19	7	0	0	0	0	0	7
≥20	58	0	1	0	0	0	59

SSR length was mostly distributed in the 12 to 20 bp range, accounting for 87.04% (4035 SSRs) of the total SSRs, followed by 21–99 bp (562 SSRs, 12.12%). There were 39 SSRs longer than 100 bp.

**Figure 6:**
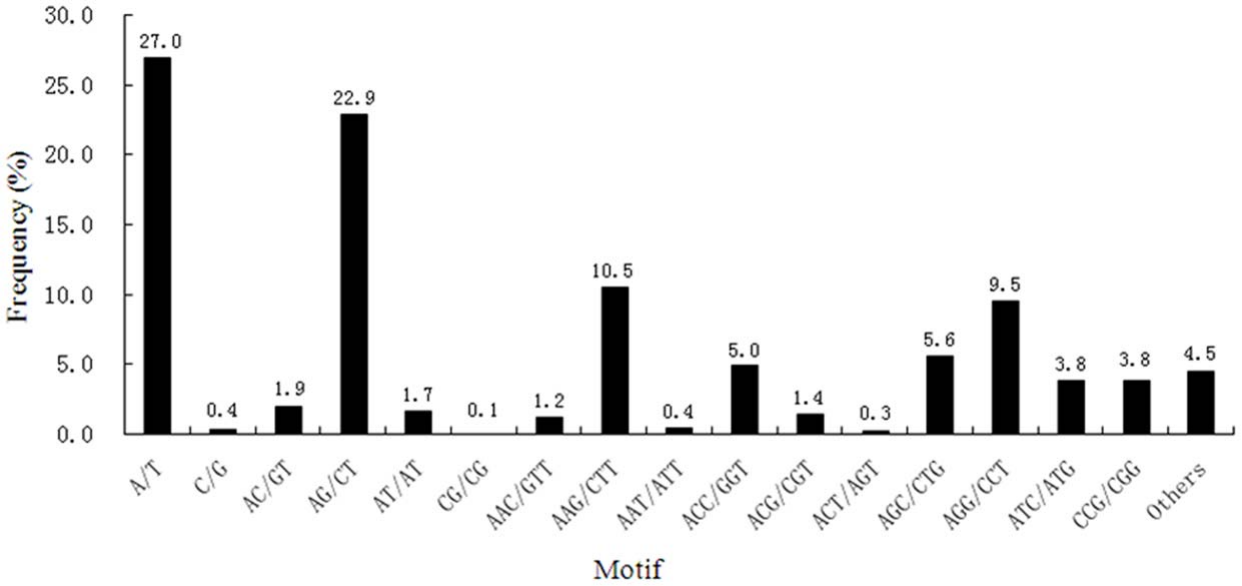
Frequency distribution of SSRs based on motif sequence types.

Within the SSRs, 119 motif sequence types were identified, of which mono-, di-, tri-, tetra-, penta- and hexa-nucleotide repeats had 2, 4, 10, 11, 31 and 58 types, respectively. The most abundant motif detected in the SSRs was the A/T mono- nucleotide repeat (1,296, 27.0%), followed by the motifs AG/CT (1,100, 22.92%), AAG/CTT (505, 10.52%), AGG/CCT (458, 9.54%), AGC/CTG (269, 5.60%), ACC/GGT (239, 4.98%), ATC/ATG (183,3.81%) and CCG/CGG (183,3.81%), AC/GT (93, 1.94%), AT/AT(80, 1.67%), ACG/CGT (68,1.42%), AAC/GTT (56, 1.17%), AAT/ATT (20, 0.42%), ACT/AGT (13, 0.27%), and CG/CG (5, 0.10%). The remaining 215 types of motifs accounted for 4.48% in total (Fig. 6).

### Identification of polymorphic markers

Fifty primer pairs (designated HM_1–HM_50) were randomly selected from the microsatellites, excluding mono-nucleotide repeats motif, to evaluate their applicability and the polymorphism across 30 individuals of *H. cordata* (Table S3). 45 of the 50 primer pairs successfully amplified fragments. Among the 48 successful primer pairs, 43 produced amplicons of expected size and 5 generated PCR fragments longer than expected. The majority of the 43 microsatellite loci showed allelic polymorphism. The number of alleles per locus varied from three to 10 (mean: 5.74). The observed heterozygosity values varied from 0.40 to 1.00 with an average of 0.83, while the expected values varied from 0.59 to 0.96 with an average of 0.78. Polymorphism information content (PIC) values ranged from 0.49 to 0.96 (mean: 0.72) (Table 5).

**Table 5:** Characterization of 43 SSRs in H. cordata.

Primer	SSRs	Forward primer (5′-3′)	Reverse primer (5′-3′)	No. of alleles	Observed heterozygosity (*Ho*)	Expected heterozygosity (*He*)	Polymorphism information content (*PIC*)
HM_1	(TGA)5	GAAGCTTAAGGAGGTAGAGGCTG	CAGTAAGTTGTGTCCAAAGGTGC	3	1.00	0.59	0.49
HM_2	(AGA)6	GAAGGACTGCAAGAAACTCTGAA	CCCCATCTTCTGTCTCTTCTCTT	7	1.00	0. 86	0.84
HM_3	(AC)7	AAACATGCATTGTCACATACAGC	AGAGCTCGCGAAATATACTGTTG	6	0.89	0.93	0.93
HM_4	(GGA)5	CGACGACGATGAGACGGT	GAGCCCACCCCATCTTTC	4	1.00	0.71	0.63
HM_5	(AG)10	AAGGGGAGAGAAAGAAAGAGAGC	CCATAATAAAGCTCAATGCTGCT	6	1.00	0.84	0.79
HM_6	(CTC)5	CTACCAAACCCCTCTCCAAAAT	GCAGCAGCAGTAGAGGATTTG	8	0.60	0.79	0.68
HM_7	(GCT)5	CTCGTTCGGCAAACTGCT	CGCGTATTATGAGCAGGATTACT	5	0.91	0.90	0.89
HM_8	(TCC)5	CAGTCCTGGTGGACATACTTGAT	CAATCGATGATGAGGAAGAAAAC	4	1.00	0.73	0.66
HM_9	(TGA)6	CTGCTGAGTCTGCATTAATTCCT	CAGCAGCTTCAGATACCTCAGA	7	1.00	0.79	0.75
HM_10	(AG)6	TATGGTGATAATAATTCCGGTCG	TTCTCCCAAATGATGGATGATAA	8	0.67	0.80	0.72
HM_11	(TGG)5	GGTGCAGGTGTTGATGTCCT	CAAGGAGAAGTTCGAGGAGGT	10	0.65	0.82	0.73
HM_12	(TTC)6	GAGTCTTCCATTTCTTTTGCTGA	GAGTGATGTGGAATGCTCTCTTC	4	1.00	0.76	0.70
HM_13	(TGA)7	GAGCATGACCTTAAAAAGGATGA	TAGACAAAAAGAATACCATCGGC	4	1.00	0.75	0.68
HM_14	(TA)7	CATGTCAACGTCATCTGTAGCAT	ATTTGGGTAGGGTACTGGAGCTA	5	0.80	0.69	0.65
HM_15	(CT)6	ACCATCTCCTCCGTTCCAC	GGAAGTAAAGATCGAGGAGGTCT	8	1.00	0.89	0.84
HM_16	(GCG)6	AGTGATACCTCCTTCTTCCGCTA	AGCAACAGGTGACAACGTACC	7	0.63	0.82	0.65
HM_17	(TTC)5	GAGTGAAAGTGTTTGCTTTGGAG	CCAACAGAACCAAATAAAACCAA	6	1.00	0.85	0.80
HM_18	(ACT)5	AATTCGAACGCGTAATCATCTT	CTAACTTCGTCGAATTGGGTCTT	6	0.54	0.65	0.63
HM_19	(CCA)5	CCTCCTCCTCTTATGGGGG	GTGGTGGCGGTCTATTTTCTAA	4	0.58	0.96	0.96
HM_20	(TCA)5	TCAGTATTGAATTTTGTCTCCCC	CTTGTTAAACGAGTGATATCGGG	7	0.88	0.79	0.78
HM_21	(GA)8	AAATAGAGTTACAGGCCCCAAAA	CTTTGTACTTTTGCGTCCACTTT	8	0.64	0.83	0.76
HM_22	(TTC)5	TTCTGCTTCTTCCCCTTCTTATT	GCTTTTACAGAGACCTCTTGTGC	4	0.50	0.67	0.62
HM_23	(TCT)6	GCATTACAAGAACCCAAACACAT	ACTACTGGTTCTAAGCGAGGAGC	4	1.00	0.68	0.60
HM_24	(GAA)5	AAATTTTAACAGCAATTCCTCCG	TCCTTCTCTGATCTAGGGTTTCC	4	1.00	0.65	0.57
HM_25	(GCG)6	AAGGAGTCCCTGGATACTGCTAC	ACCACAAAACGAACGGATTC	7	0.40	0.61	0.52
HM_26	(CT)7	TTCTTCGCTTCTAGATTCCCTTT	TCTAGCCAAAGTCTTCCTTCCTT	8	0.67	0.76	0.65
HM_27	(GA)8	ATCAAAGCTGCAAGCTTATATCG	TTGTTCACCACTGGAATCACA	5	0.60	0.80	0.73
HM_28	(GGA)5	GGTCTACGGCAAACAGACTAGC	CGATCCTCCAACTCCAAAAA	7	0.59	0.80	0.71
HM_29	(AT)8	CTAGCAGACTGGATGTTGGTTTT	GTAAGGCGCATAATACTTGTTGC	4	1.00	0.73	0.66
HM_30	(GCT)5	AGATCCCCACCCTTTCTTGTAT	ATGACTTGATGAAAAAGAGGCAA	4	1.00	0.75	0.68
HM_31	(GCCCCA)4	GGCATTGCCTATAGAAGAAGTATCA	CATTGTTCCACCTGGGTAAGAT	5	0.75	0.63	0.59
HM_32	(TGT)7	GTACGAAGAGGAGGAATTGGG	TCAGAAAAGAAAAACCAGACCAA	8	1.00	0.87	0.82
HM_33	(TCC)5	GTCAGATCGAGGAGCGTAAATC	CCTCCTGATGGAATGAAGATG	6	0.68	0.78	0.67
HM_34	(GAA)7	GACGCAAGGTAACCCTCAATAAT	ATTGCTTTCACTCTGGTTGTCAT	5	0.52	0.71	0.64
HM_35	(GGCGAT)7	GAGGGAAGAAGGCGATCAG	CTGTCTTCTCGAGGACTGCTCT	3	1.00	0.63	0.54
HM_36	(TC)9	ATTCCCTTCTTCTCTGCCTCTC	CGTGCTCAAAGTAGCAGAATCA	3	0.86	0.91	0.90
HM_37	(AGA)5	CCATAAATACATCACCTCGATCC	CCAGCAGAGTTCAACTTGTTCTT	5	0.78	0.80	0.77
HM_38	(GA)7	ATCTTATCCACAACAAGAAGCCA	AAGCTCATGAACATGACACACAG	3	0.91	0.90	0.89
HM_39	(TC)6	CTCAGGAAAAGTAACACTGGCAT	GAGACTTGTCTCCTGCATCAACT	7	1.00	0.85	0.80
HM_40	(GA)9	AGCCTTCAGGATTACTTCTCCAC	AAGATGTCTTCAATCTTCACCCA	5	0.80	0.69	0.65
HM_41	(ACC)6	GCATTTTGTTTGGATTGACAAAG	TGGAGTGCTTTTTAGCAAATTGT	6	1.00	0.79	0.75
HM_42	(TAT)5	GAAGAAGATGATGCTGATGGTTC	ATTCTGTGCAGCTAGTTACCACC	9	0.90	0.89	0.83
HM_43	(CAC)7	CTGTTGAGGCTGAGGATATTGAG	AAACGCGTCAAAGAAGAACATC	8	1.00	0.86	0.81
Mean				5.74	0.83	0.78	0.72

## Discussion

Next generation sequencing technologies provide a low cost, labor saving and rapid means of transcriptome sequencing and characterization [44], which enables various functional genomic studies on an organism. Although the 454 Life Sciences (Roche) technology is often used for transcriptome analysis of non-model organisms, it is more expensive than the Illumina technology [45]. De novo assembly of short reads without a known reference is considered difficult [46], but de novo assembly of transcriptomes using short reads has received attention [47]. In this study, we demonstrated a strategy for de novo assembly of a transcriptome using short reads for a non-model medicinal plant, *H. cordata*, for which sequence data is very limited in the public databases at present. We showed that assembly program parameters and sequence quality have a significant effect on the assembly output. Although the length of contigs were often less than 500 bp, the Illumina sequencing solution was reliable. such as the average contig size of sesame was less than 200 bp [48], whitefly was only 40 bp [49], sweetpotato was 202 bp[50]. Compared with these reports, the assembled contigs in this study was quite long (253 bp). This suggested that the coverage was relatively high. Greater N50 and average lengths are considered indicative of a better assembly. Here, the N50 length of the unigenes was 1,051 bp and the average length was 679 bp, which suggests that the relatively short reads from Illumina paired-end sequencing for this non-model organism have been effectively and accurately assembled.

Illumina sequencing yielded 56.67 million paired-end reads for *H. cordata*. The 63,954 unigenes produced here may be useful for further research into *H. cordata* functional genomics. Of the *H. cordata* unigenes, 39,982 (62.52%) showed homology to sequences in the Nr database. Comparatively, in *Epimedium sagittatum* [51], whitefly [49], sweet potato [50] and sesame [48], only 38.50%, 16.20%, 46.21% and 54.03% of the unigenes, respectively, had homologs in the Nr database. The average unigene length in our database was 679 bp, compared with 246, 266, 581 and 629 bp, respectively, in the four studies mentioned above. The higher percentage of hits found in this study was partially a result of the increased number of long sequences in our unigene database; the results for whitefly [49] and sesame [48] support this conclusion. Homologs in other species were not found for 18.3% of the unique sequences. Specifically, only 29.28% of the unigenes shorter than 300 bp showed matches, meaning that 70.72% produced no hits (Fig. 3). These shorter sequences may lack a characterized protein domain, or they may contain a known protein domain but the query sequence is too short to show sequence matches, resulting in false-negative results. Additionally, little genomic and transcriptomic information is currently available for *H. cordata*, and consequently, many *H. cordata* lineage-specific genes might not be included in current databases.

Both gene annotation and KEGG pathway analyses are useful for predicting potential genes and their functions at a whole-transcriptome level. In the *H. cordata* transcriptome, the predominant gene clusters are involved in the cellular process and metabolic process categories of the biological process GO domain, the cell and cell part categories of the cellular component domain, and antioxidant activity and binding categories of the molecular function domain. Similar results were found in sesame [51] and whitefly [49]. However, in the chickpea transcriptome, the sequences were found to be mainly involved in protein metabolism (biological process) and transferase activity (molecular function) [52]. This suggests remarkable differences among different species of plants.

KEGG analysis showed that 24,434 sequences were involved in 128 known metabolic or signaling pathways, including endocytosis, plant hormone signal transduction and plant-pathogen interaction. *H. cordata* is one of the most important medicinal plants and is rich in secondary metabolites, which makes it a very important target for genomic studies. In this study, 2,448 (10.02%) sequences of *H. cordata* were associated with biosynthesis of secondary metabolites (Table 2). These results may be useful for further investigation of gene function in the future.

The large number of sequences generated for *H. cordata* in this study for the first time provides valuable sequence information at the transcriptomic level for screening of novel functional genes, or for investigation of molecular mechanisms.

SSR markers play an important role in genetic diversity research, population genetics, linkage mapping, comparative genomics, and association analysis [38], [53]. Previously, genetic diversity analysis of *H. cordata* germplasm was restricted to AFLP [37], ISSR [4], PCR-RFLP [30] and RAPD markers [31]. One of the main reasons for this was the lack of a genome sequence or transcriptome information for *H. cordata*. Our results have resolved this problem and enabled development of SSR markers for this species. In the present study, 4,800 perfect microsatellites exceeding 12 bp were identified from the *H. cordata* dataset, and 119 motif sequence types were identified. If mono-nucleotide repeats were excluded, di-nucleotide repeats were the most abundant type, followed by tri- nucleotide repeats, which is consistent with previous reports [54]–[57]. The fact that the most abundant di- and tri-nucleotide motifs were AG/TC and AAG/TTC, respectively, was also coincident with previous reports on other species of plants [54]–[56].

In this study, 48 (96%) of the primer pairs designed from the unigenes successfully yielded high-quality amplicons. These results suggested that the unigenes were suitable for specific primer design, that the assembled unigenes were of high quality, and that the SSRs identified from our dataset could be useful in the future. Five primer pairs produced products that deviated from the expected size, which might have been caused by the presence of introns [51], [58], [59], large insertions or repeat number variations, or a lack of specificity [51]. The failure of two primer pairs to produce amplicons might have been because of the primer(s) being located across splice sites, large introns, chimeric primer(s), poor-quality sequences, or assembly errors [51], [59].

The 43 primer pairs in our dataset were used to investigate polymorphisms in 30 individuals of *H. cordata* from 15 populations located across the natural distribution of the species in 13 provinces of China. The results indicated that the level of polymorphism was relatively high, which was also coincident with previous reports using ISSR [4], RAPD [31] and RAMP markers [60]. Since we identified other SSRs in our dataset, more PCR primers could be developed that would be very useful in germplasm polymorphism assessment, quantitative trait loci mapping [61], comparative genomics [62], functional genomics and proteomics studies.

## Conclusions

In this study, we have analyzed the transcriptome of *H. cordata* using high-throughput Illumina paired-end sequencing. We obtained 39,982 sequences and demonstrated some important features of the *H. cordata* transcriptome through gene annotation and KEGG pathway analysis. In addition, we identified reliable genetic markers in the form of 4,800 SSRs. Fifty primer pairs were randomly selected to detect polymorphism among 30 *H. cordata* accessions, and 43 (86%) of these primer pairs successfully amplified fragments, revealing abundant polymorphisms. The SSR markers developed in this study can be used for construction of high-resolution genetic linkage maps and to perform gene-based association analyses in *H. cordata*. This is the first application of Illumina paired-end sequencing technology to investigate the whole transcriptome of *H. cordata* and to assemble RNA-seq reads without a reference genome. This study will provide useful resources and markers for functional genomics and proteomics research on *H. cordata* in future.

## Materials and Methods

### Plant materials and RNA extraction

**Figure 7:**
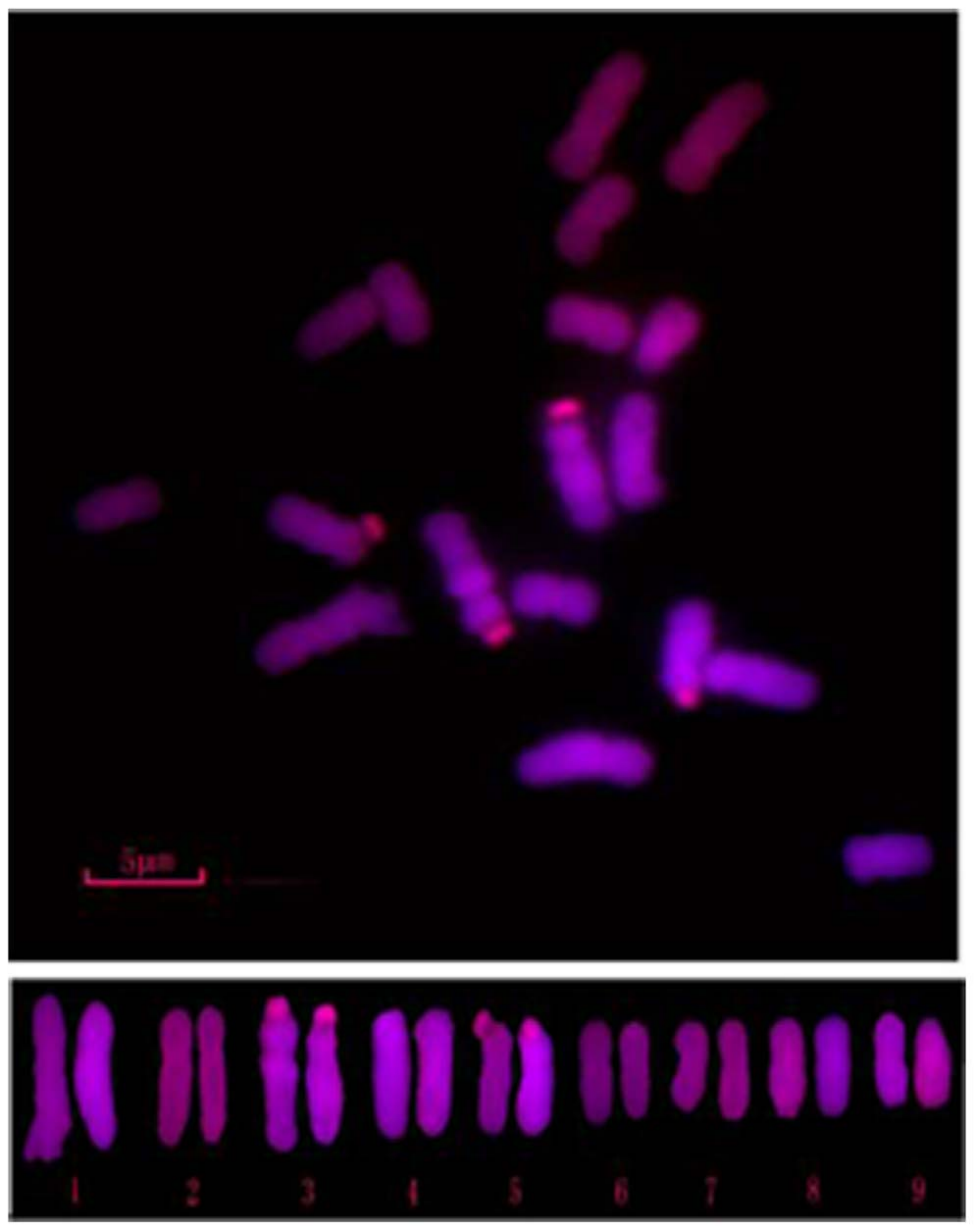
The number of chromosomes and karyotype of *H. cordata.*

*H. cordata*, whose seeds came from national forest park of Zhong Po Mountain in Huaihua City of Hunan Province, was grown in the experimental station of the Department of Life Sciences, Huaihua University, Huaihua, China. The individuals have 18 chromosomes and diploid karyotype (Fig.7). Flower, leaf, stem and rhizome tissues were harvested 14 weeks post planting,because *H. cordata* was planted in spring, and their flowers began to open after 14 weeks. All of the samples were immediately frozen in liquid nitrogen and stored at −80°C until use.

Total RNA was isolated using the TRIzol reagent according to the manufacturer's instructions (Invitrogen, Carlsbad, CA, USA). It was treated with RNase-free DNase I (Worthington, Lakewood, CO, USA) for 30 min at 37°C to remove any residual DNA. The quality and quantity of each RNA sample was determined by biophotometer (Eppendorf, Germany). Only those RNA samples with an A_260_:A_280_ ratio from 1.9 to 2.1 and an A_260_:A_230_ ratio from 2.0 to 2.5 were used for the analysis. A total of 20 µg of RNA was equally pooled from the four different tissues for cDNA library preparation.

### cDNA library construction and sequencing

Beads with oligo(dT) were used to isolate poly(A) mRNA after total RNA was collected from the samples. Fragmentation buffer was added to disrupt the mRNA into short fragments. Taking these short fragments as templates, random hexamer primers were used to synthesize first-strand cDNA. Second-strand cDNA was then synthesized using buffer, dNTPs, RNase H and DNA polymerase I. The short fragments were purified with a QiaQuick PCR extraction kit and resolved with EB buffer for end reparation and A tailing. The short fragments were then connected with sequencing adapters. After agarose gel electrophoresis, suitable fragments were selected for PCR amplification as templates. The cDNA library was sequenced on an Illumina HiSeq2000 sequencing platform.

### Data filtering and de novo assembly

The raw reads were cleaned by removing adapter and low-quality sequences, because sequencing errors can create difficulties for the short-read assembly algorithm. We therefore carried out a stringent filtering process. Firstly, we discarded all reads with adaptor contamination. Secondly, we ruled out low-quality reads with ambiguous sequences “N”. Thirdly, the reads with more than 10% Q<20 bases were also removed. De novo transcriptome assembly was carried out with the short reads assembly program in the Trinity software(Release-20120608) [42]. Contigs were created by combining reads that had a certain length of overlap. The reads were then mapped back to the contigs; with paired-end reads we were able to detect contigs from the same transcript as well as the distances between the contigs. The contigs were connected using the Trinity software to get sequences that could not be extended at either end. Such sequences were defined as unigenes. These unigenes were further processed by sequence splicing and redundancy removal using the TGICL software(Version 2.1) [63] to acquire non-redundant unigenes that were as long as possible. After gene family clustering, the unigenes could be divided to two classes. One was clusters, including unigenes that were >70% similar to each other; the other was singletons. In the final step, the sequence direction of the unigenes was determined.

### Function annotation

The unigenes were first aligned to sequences in the NCBI Nr and Swiss-Prot protein databases with an E-value <10^−5^ using BLASTx. Unigenes that did not have homologs in the databases were scanned using ESTScan (Version3.0.2) [43]. Blast2GO(Version 2.5.0) [64] was used to obtain GO annotations for the unigenes based on BLASTx hits against the NCBI Nr database with an E-value threshold of <10^−5^. WEGO [65] was used for GO functional classification of all unigenes and to plot the distribution of the *H. cordata* gene functions. The unigene sequences were also aligned to the COG database to predict and classify their functions. Pathway assignments were carried out based on the KEGG database [66], which contains a systematic analysis of inner-cell metabolic pathways and the functions of gene products.

### Simple sequence repeat marker discovery and primer design

A microsatellite program (MISA; http://pgrc.ipkgatersleben.de/misa/) was used to identify and localize microsatellite motifs. We searched for all types of simple sequence repeats (SSRs) from mononucleotide to hexanucleotide using the following parameters: at least 12 repeats for mono-, six repeats for di-, five repeats for tri- and tetra-, and four repeats for penta- and hexa-nucleotide simple repeats. Primer pairs were designed using the software Primer 3-2.2.2. The major parameters for primer design were set as follows: primer length of 18–25 bases (optimal 21 bases), PCR product size of 80–200 bp (optimal 100–150 bp), GC content of 40–60% (optimal 50%), and DNA melting temperature of 57–64°C (optimal annealing temperature 55–59°C).

### Survey of SSR polymorphism

Thirty individuals of *H. cordata* from 15 populations located across the natural distribution of the species in 13 provinces of China (Table S3) were selected for polymorphism investigation with the SSRs. Leaf samples were collected, dried and preserved in silica gel until DNA extraction. Genomic DNA was extracted from the leaves of each individual using the CTAB protocol [67], dissolved in double distilled water, and quantified using agarose gel electrophoresis. The DNA concentration was calculated according to DNA standards. PCR amplification was performed in 16 µL reaction mixtures. Each reaction contained 0.2 µL *Taq* DNA polymerase (0.5 U/µL), 2.5 µL PCR buffer, 1.5 µL MgCl_2_ (25 mmol/L), 0.5 µL dNTPs (2.5 mmol/L), 0.4 µL each primer (10 pmol/L), 2.0 µL template DNA (50 ng/µL), and 8.5 µL sterilized H_2_O. The temperature profiles were: initial denaturation at 94°C for 3 min, 35 cycles of denaturation at 94°C for 30 s, annealing at the optimal temperature of each primer pair for 30 s, and extension at 72°C for 45 s. Final extension was at 72°C for 5 min, and then samples were held at 4°C. After PCR amplification, 6 µL aliquots of the amplified PCR products were loaded onto an 8% polyacrylamide gel. After 3–4 h of electrophoresis (250 V), the gels were stained with silver nitrate (silver staining). Perfectly amplified loci were tested for polymorphism by genotyping 30 individuals of *H. cordata*. The genetic diversity and mean allele number were calculated using Popgene version 1.32 [68]. Polymorphism information content (*PIC*) was derived according to the following formula:
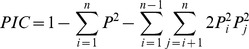
where *n* is the number of alleles at one locus; *Pi* and *Pj* are the frequencies of the *i*th and *j*th alleles at one locus; *j* = *i*+1.

## Supporting Information

Table S1 The statistics and annotations of unigenes.
(XLS)

Table S2 The KEGG pathways.
(XLSX)

Table S3 30 individuals of *H. cordata* examined in the simple sequence repeat (SSR) analysis.
(XLSX)
